# A comparative analysis of label-free liquid chromatography-mass spectrometry liver proteomic profiles highlights metabolic differences between pig breeds

**DOI:** 10.1371/journal.pone.0199649

**Published:** 2018-09-12

**Authors:** Samuele Bovo, Alessio Di Luca, Giuliano Galimberti, Stefania Dall’Olio, Luca Fontanesi

**Affiliations:** 1 Department of Agricultural and Food Sciences, University of Bologna, Bologna, Italy; 2 Department of Statistical Sciences “Paolo Fortunati”, University of Bologna, Bologna, Italy; INIA, SPAIN

## Abstract

The liver is a complex organ governing several physiological processes that define biological mechanisms affecting growth, feed efficiency and performance traits in all livestock species, including pig. Proteomics may contribute to a better understanding of the relationship between liver functions and complex production traits in pigs and to characterize this species as biomedical model. This study applied, for the first time, a label‐free liquid chromatography-mass spectrometry (LC‐MS) proteomic approach to compare the liver proteome profiles of two important heavy pig breeds, Italian Duroc and Italian Large White. Liver specimens were collected (after slaughtering) from performance tested pigs of these two breeds, raised in standard conditions. The label‐free LC‐MS method captured a total of 501 proteins of which 200 were subsequently considered in the between breeds comparison. A statistical pipeline based on the sparse Partial Least Squares Discriminant Analysis (sPLS-DA), coupled with stability and significance tests, was applied for the identification of up or down regulated proteins between breeds. This analysis revealed a total of 25 proteins clearly separating Italian Duroc and Italian Large White pigs. Among the top proteins differentiating the two breeds, 3-ketoacyl-CoA thiolase, mitochondrial (ACAA2) and histone H2B type 2-F (HIST2H2BF) were up-regulated in Italian Duroc pigs and carboxylesterase 3 (CES3) and ketohexokinase (KHK) were up-regulated in Italian Large White pigs. Fatty acid synthase (FASN), involved in fatty acid metabolism and encoded by a gene located in a QTL region for fatty acid composition, was up-regulated in Italian Large White pigs. The *in silico* protein interaction analysis showed that 16 of these proteins were connected in one big module. Bioinformatic functional analysis indicated that differentially expressed proteins were involved in several biological processes related to the metabolism of lipids, amino-acids, carbohydrates, cofactors and antibiotics/drugs, suggesting that these functions might distinguish Italian Duroc and Italian Large White pigs. This pilot comparative proteomic analysis of the porcine liver highlighted several biological factors that could determine the peculiar production potentials of these two heavy pig breeds, derived by their different genetic backgrounds.

## Introduction

The liver is an important metabolic organ that governs many physiological processes that define biological mechanisms leading to growth, feed efficiency and several other economically relevant traits in all livestock species, including pig. The liver is involved in lipid oxidation, is a primary source of proteins and the centre of amino acid metabolism, is responsible for the secretion of many proteins into the blood and interplays with this circulating tissue for the transfer of amino acids and energy compounds and the disposal of waste metabolites, i.e. derived by the protein degradation in the form of urea metabolism [1, 2].

The construction of liver proteome maps [3, 4] and the establishment of the Human Liver Proteome Project (HLPP) by the Human Proteome Organisation (HUPO) [5] are important resources for the study of liver function not only in humans but also in all animal species [6, 7]. Indeed, a better understanding of the metabolic processes being undertaken in the hepatocytes will eventually be a step forward in managing animal growth and feed efficiency. In this context, proteomics can help to unravel the variations in the metabolic pathways of the hepatocytes in healthy and diseased animals and to identify biomarkers that could be applied in breeding programmes. Proteomic information in livestock species can be also useful to define animal models to better characterize liver physiology and related diseases [8, 9].

Despite these key roles and envisaged potential applications, the interest of the animal science sector on liver proteomics has been slowly increasing over the last years. For example, Timperio *et al*. [10] compared the cattle liver proteome profiles using traditional proteomic approaches [including two-dimensional polyacrylamide gel electrophoresis (2DE) and mass spectrometry (MS)], assuming that differences could be imputed to genetic diversity between dairy and beef breeds [10].

A first comprehensive proteomic analysis of porcine hepatic cells was achieved by Caperna *et al*. [11] who analysed three-lines hybrid pigs (Landrace × Yorkshire × Poland China). This study produced 2DE maps of cytosol and membrane fractions from hepatocytes that were subsequently characterized by MS analysis. A similar approach was used by Wang *et al*. [12] and Liu *et al*. [13] to investigate how intrauterine growth restriction affected the liver proteome in new borne and fetal pigs.

More recently, gel-free proteomic approaches have risen in popularity [14] and have been also applied in livestock. Tang *et al*. [15] investigated the heat stress response in broiler liver using a gel-free method, i.e. sequential window acquisition of all theoretical spectra (SWATH)-MS, reaching a good resolution that was able to quantify about 2,400 proteins. One tenth of these proteins was differentially expressed between the controls and the heat stressed group. Other MS based proteomics methods, such as label-free liquid chromatography (LC)-MS, have become more frequently adopted for quantifying protein expression and comparing different samples. This method can also give the possibility to better investigate hydrophobic proteins with low or high molecular weights that are quite challenging to be analysed using traditional proteomic approaches [14].

It is well known that pig breeds and lines differ in their potentials for many production and economically relevant traits (like growth rate, feed efficiency, carcass and meat quality traits, reproduction performances and rusticity) that might affect their use or choice in various production systems and breeding programmes. These traits can be considered as external traits or end phenotypes and are the outcome of complex biological processes and interactions. Therefore, a more detailed description of these phenotypes can be obtained only adding intermediate or molecular information, which can dissect the biological mechanisms underlying their final expression [16]. This intermediate level that links the genetic layer (defined by the genetic variability) and the external phenotypes (i.e. production traits) includes internal phenotypes such as metabolite and protein quantitative and qualitative profiles [17]. Recently, comparative metabolomic analyses of plasma and serum of two Italian heavy pig breeds (Italian Duroc and Italian Large White) have identified biomarkers that might explain the metabolic differences between these breeds [18]. Italian Duroc and Italian Large White are two heavy pig breeds, selected under the national pig breeding program, that differ for several economically important traits (e.g. growth rate, feed efficiency, intermuscular fat deposition, meat quality, rusticity and reproduction traits) [19–21]. These breeds are commonly used in crossbreeding plans to produce final pigs, for the production of high quality dry-cured hams [19–21].

This study characterized and compared the liver proteomic profiles of Italian Duroc and Italian Large White pigs using a quantitative label-free LC-MS proteomic approach. The final aim was to identify intermediate phenotypes that might capture biological differences between these two porcine genetic types and then inform the biological mechanisms underlying their different production performances.

## Materials and methods

All animals used in this study were kept according to the Italian and European legislations for pig production. All procedures described here were in compliance with Italian and European Union regulations for animal care and slaughter. Animals were slaughtered in a commercial abattoir following standard procedures. Animals were not fasted for the purpose of this study. Fasting was part of the standard pre-slaughtering procedures. Animals were raised in an approved performance tested structure. Pigs were not raised or treated in any way for the purpose of this study and for this reason no other ethical statement is needed.

### Samples

Five Italian Duroc gilts and five Italian Large White gilts were included in this study. Animals did not have common grandparents. Gilts from both pig breeds were performance-tested at the Test Station of the National Pig Breeder Association (ANAS). Performance evaluation started when the pigs were 30 to 45 days of age and ended when the animals reached about 155 ± 5 kg live weight. Details of performance testing system in Italian heavy pigs of these two breeds are reported in [22–24].

The management of all pigs included in this study with regard to housing, feeding, handling and transportation was the same. All animals were fed with the same standard commercial feed for fattening pigs under the production rules of the Parma and San Daniele dry-cured ham consortia. After a fasting period of 12 h, all animals, which were nine months old (and weighting 155 ± 5 kg), were transported in the morning in a commercial abattoir, where they were electrically stunned and then slaughtered under controlled conditions. Then, after slaughtering, liver specimens were collected from the extreme end of right lobe, placed in a labelled tube, snap frozen in liquid nitrogen and then stored at −80°C until they were analysed.

### Protein extraction and digestion

Protein extraction and digestion were performed according to a slightly modified Filter Aided Sample Preparation (FASP) protocol to improve sensitivity, recovery and proteomic coverage for processed samples [25]. Briefly, proteins were extracted from approximately 100 mg of liver tissue homogenised in 1 mL of extraction buffer [0.1% SDS (Affymetrix/Thermo Fisher Scientific, USA), 100 mM Tris/HCl (Affymetrix/Thermo Fisher Scientific, USA) pH 7.6, 10 mM DTT (Affymetrix/Thermo Fisher Scientific, USA)]. Liver samples were homogenised for 2 min using a homogenizer (G50 Tissue Grinder, Coyote Bioscience, Inc., China) in an Eppendorf tube immersed into liquid nitrogen. The lysates were vortexed at room temperature for 3 min and then centrifuged at 16,000 × r.c.f. at 4°C for 5 min. Samples were heated at 56°C for 30 min and then centrifuged at 16,000 × r.c.f. at 20°C for 20 min. The supernatants were transferred into new labelled tubes, were mixed, aliquoted and stored at -20°C until usage. Qubit™ Fluorometric Quantitation was used to determine the protein concentration according to the manufacturer’s instructions (Affymetrix/Thermo Fisher Scientific, USA).

Protein samples were reduced and alkylated with dithiothreitol (DTT) and iodoacetamide (IAA; Sigma-Aldrich/Merck, USA) and then digested with trypsin according to the FASP method [25]. Samples were then purified from any contaminants using Pierce C18 Spin Columns (Affymetrix/Thermo Fisher Scientific, USA), vacuum dried and stored at -20°C. All 10 peptide samples (five from Italian Duroc and five from Italian Large White pigs) were collected for MS analysis.

### Mass spectrometry analysis

Dry peptides from each sample were resuspended in 25 μL of a mixture of water:acetonitrile:formic acid 97:3:2 and sonicated for 10 min at room temperature, then centrifuged at 12,100 x r.c.f. for 10 min. Mass spectrometry analyses were performed on an ESI-Q-TOF Accurate-Mass spectrometer (G6520A, Agilent Technologies, Santa Clara, CA, USA), controlled by MassHunter software, Agilent (v. B.04.00) (https://www.agilent.com/en/products/software-informatics/masshunter-suite/masshunter/masshunter-software) and interfaced with a CHIP-cube to an Agilent 1200 nano-pump (Agilent Technologies, Santa Clara, CA, USA).

Chromatographic separation was performed on a high-capacity loading chip (Agilent Technologies) with a 75 μm internal diameter (I.D.), 150 mm, 300 Å C18 column, prior to a desalting step through a 500 nL trap column. The injected samples (8 μL) were loaded onto the trap column with a 4 μL/min 0.1% (v/v) formic acid (FA):acetonitrile (ACN) (98:2 v/v) phase flow. After 3 min, the precolumn was switched in-line with the nanoflow pump (400 nL/min, phase A: water:ACN:FA (96.9:3:0.1 v/v/v), phase B: ACN:water:FA 94.5:5:0.1 v/v/v), equilibrated in 1% (v/v) B. The peptides were eluted from the reverse phase (RP) column through the following gradient: 1% for one min, 1->5% B in 7 min, 5->30% B over a period of 142 min, 30–60% B in 20 min, 60->90% B in 0.1 min, then held at 90% B for 8 min, and switched back to 1% B for column reconditioning, for a total runtime of 190 min. Eight μL of each samples were run twice; analytical controls (a mix of baker’s yeast enolase and bovine serum albumin tryptic digests) were run daily to monitor chromatographic performances. Ions were formed in a nano-ESI source, operated in positive mode, 1860 V capillary voltage, with the source gas heated at 350°C and at a 5 L/min flow. Fragmentor was set to 160 V, skimmer lens operated at 65 V. Centroided MS and MS^2^ spectra were recorded from 250 to 1700 m/z and 70 to 1700 m/z, respectively, at scan rates of 8 and 3 Hz. The six most intense multi-charged ions were selected for MS^2^ nitrogen-promoted collision-induced dissociation. The collision energy was calculated according to the following expression:

$$CE(V)=3.6∙\frac{m/z}{100}-3$$

A precursor active exclusion of 0.22 min was set, and the detector was operated at 2 GHz in extended dynamic range mode. Mass spectra were automatically recalibrated with two reference mass ions.

### Label-free quantitative profiling

Raw MS data were converted to MASCOT (www.matrixscience.com) generic file (mgf) using MassHunter Qualitative Analysis (v. B.05.00, Agilent Technologies). Then data were used for peptide identification with MASCOT (version 2.4, Matrix Science, London, UK) searched against an implemented version of the Pig PeptideAtlas resource (http://www.peptideatlas.org/) [26], including contaminant protein sequences as retrieved from the cRAP v.1.0 resource (http://www.thegpm.org/crap/). The search parameters used were as follow: 40 ppm precursor tolerance, 0.1 Da fragment mass error allowed, two missed cleavage allowed for trypsin, carbamidomethyl as a fixed modifier of cysteine residues, asparagine and glutamine deamidation and methionine oxidation as variable modification. The peptide identification results were imported into an in-house Trans-Proteomic Pipeline server (TPP, v. 5.1.0) [27], rescored and validated with PeptideProphet [28]. Protein inference was then achieved by ProteinProphet [29]. As last step, relative label-free quantification was performed with Skyline v.4.1 [30] using the rescored search results [Protein probability ≥0.9, corresponding to a global false discovery rate (FDR) ≤0.1]. PepXML files and raw mzXML data were imported in Skyline and: (i) the protein database was filtered by retaining only the proteins with a ProteinProphet score ≥ 0.9 and (ii) MS^1^ data were correlated to the corresponding peptide identification, matches were filtered (idotP≥0.9).

Data were exported and analyzed with InfernoRDN v.1.1.6556.25534 (https://omics.pnl.gov/software/InfernoRDN; [31]). Data were Log2 transformed and normalized with the central tendency adjustment method. Peptide measurements were then rolled up to corresponding protein abundances through the Zrollup method. In the ZRollup procedure a scaling method similar to z-scores is applied first to peptides that originate from a single protein and then the scaled peptide measures are averaged to obtain a relative protein abundance measure [31]. Differences in protein abundance between Italian Duroc and Italian Large White pigs were investigated as described below. Proteins were considered only if identified with more than one peptide.

The mass spectrometry proteomics data have been deposited to the ProteomeXchange Consortium via the PRIDE (http://www.ebi.ac.uk/pride; [32]) partner repository with the dataset identifier PXD009771 and project DOI: 10.6019/PXD009771.

### Analyses

#### Multivariate statistical analysis

Differentially abundant proteins were detected by applying the multivariate approach of sparse Partial Least Squares Discriminant Analysis (sPLS-DA) [33] coupled with the validation procedure detailed in Bovo *et al*. [18, 34]. sPLS-DA is a multivariate technique used in classification and discrimination problems especially when variables are highly correlated [35]. Briefly, breed was modeled as response variable (Italian Duroc = 0, Italian Large White = 1) and proteins as predictors. The sPLS-DA penalization coefficient *eta* (ranging from 0.1 to 0.9) and the number of hidden components *K* (ranging from 1 to 5) were automatically selected by an internal 4-fold cross-validation procedure (4CV). The selected proteins were then assessed through a stability and significance test [18]. The stability test was based on a Leave One Out (LOO) procedure coupled with a permutation test. For this purpose, 1,000 artificial datasets were obtained by randomly permuting the breed trait values. While the stability test is aimed at evaluating the frequency of selection of a protein in the original dataset against the permuted ones, the significance test evaluates the regression coefficient (β). These two tests estimate the probability (*P*) that the selection of a given protein inside the dataset is due to chance or to a particular structure of the dataset. Proteins having a *P* < 0.10 (with the sign of the regression coefficient that matched the fold change ratio) were declared stable and significantly related to breed. Analyses were performed in R v. 3.0.2 [36] by using the “spls” package (function “cv.splsda” and “splsda”). Scatter plot of the first two components was drawn (each point represents an individual sample).

#### Functional and protein network analyses and relation to genomic information

Functional interpretation of differentially abundant proteins was carried out in Cytoscape (http://www.cytoscape.org/) [37] using the plug-in ClueGO (http://www.ici.upmc.fr/cluego/) [38]. Gene enrichment analysis was carried out over the Gene Ontology (GO)–Biological Process (BP) branch (release data: May 2018) by setting the following parameters: (i) GO hierarchy level from 3 to 20; (ii) minimum number of genes per GO term equal to 2; (iii) minimum of input genes associated to the functional term greater than 2%; (iv) GO term network connectivity (Kappa score) equal to 0.70 and right-sided hypergeometric test. These adjustments were applied to maximize the process of functional association. Indeed, the tuning of the above-mentioned parameters allows to over-represent processes and functions very specific (the lowest levels of the GO hierarchy) rather than terms that are general and uninformative (the highest levels of the GO hierarchy). Moreover, similar processes and functions are clustered in functional groups representing closely related terms. The analysis made use of *Sus scrofa* specific functional annotations. The other parameters were kept with default values. GO:BP terms with a Benjamini–Hochberg corrected *p*-value < 0.05 were considered statistically over-represented. A pathway enrichment analysis was separately carried out, with ClueGO, over the KEGG pathway database (release data: May 2018).

The *in silico* Protein-Protein Interaction (PPI) analysis of differentially abundant proteins identified in the breed comparison was obtained using STRING v. 10.5 database (https://string-db.org/) [39]. STRING is a web resource providing uniquely comprehensive coverage of experimental and predicted interaction information. The analysis was carried out considering the *Sus scrofa* specific interactome. Only interactions having a STRING combined score > 0.4 were considered (i.e. medium confidence). Network indices such as the number of nodes and edges, the average node degree (average no. of connections), the expected number of edges and the PPI enrichment *p*-value were computed. The expected number of edges gives how many edges would be expected if the nodes were selected at random. A small PPI enrichment *p*-value indicates that nodes are not random and that the observed number of edges is significant (https://string-db.org/).

The Pig Quantitative Trait Locus Database (Pig QTLdb release 35) [40] was used to explore possible links between up or down-regulated proteins and genomic QTLs for economically relevant traits and parameters that could be affected by genes encoding these proteins. The whole set of 27,465 QTLs/associations was downloaded and their genome coordinates were compared with genome coordinates of the genes encoding differentially abundant proteins observed in this study. Borders of the considered genomic regions were defined by the gene coordinates ± 50 kbp. QTL relationships were filtered by manually curating functional relationships of the genes encoding these proteins.

## Results

### Label-free proteomic data of porcine liver samples

**Fig 1:**
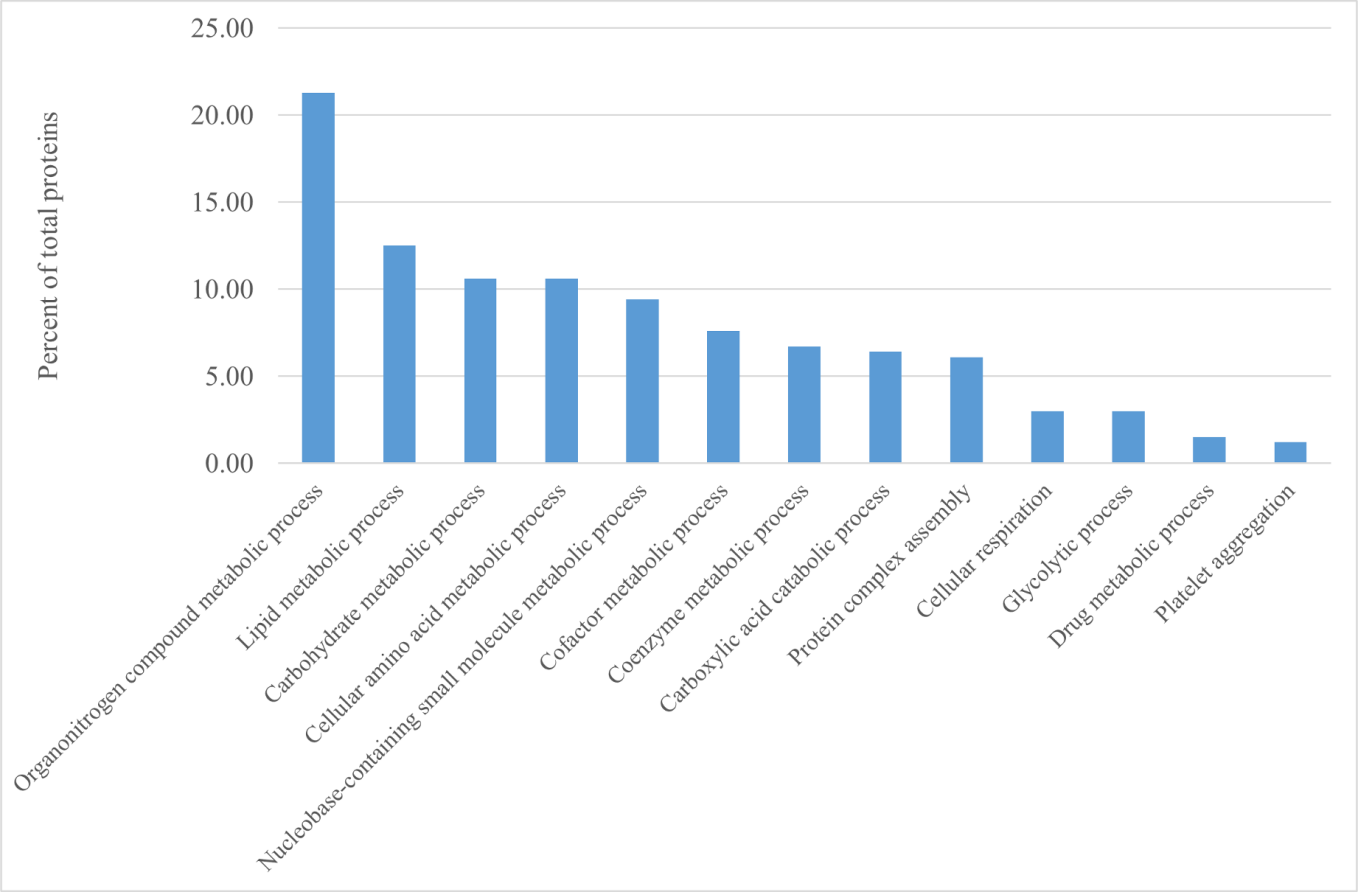
Percentage of liver proteins (over all 329 identified proteins; S1 Table) grouped according to different biological processes.

Label-free proteomic analysis was performed from proteins extracted from the liver of pigs of two heavy pig breeds, Italian Duroc and Italian Large White. The analysis of the Italian Duroc liver specimens identified a total of 1,696 peptides belonging to 467 proteins, with an average of 3.6 peptides per protein. The analysis in the Italian Large White liver samples identified 1,253 peptides related to 350 proteins, with an average of 3.6 peptides per protein. Combining these two datasets, this study identified a total of 1,873 peptides related to 501 proteins (average of 3.7 peptides per protein), that were then re-defined using stringent parameters in the InfernoRDN analysis, obtaining 329 unique proteins (S1 Table). These proteins were included in a total of 13 GO:Biological Processes (Fig 1), four of which (organonitrogen compound metabolic process, lipid metabolic process, carbohydrate metabolic process and cellular amino acid metabolic process) accounted each for more than 10% of the total listed proteins.

Only proteins having at least two supporting peptides were kept and subsequently used in the study. Bioinformatic analyses were carried out on a final dataset counting 200 proteins (including a total of 1,041 peptides) identified with high confidence and subsequently quantified. The full list of the 200 proteins analysed in this study, as well as the information on the identified peptides, is shown in the S2 Table.

### Comparative analysis of breed derived proteomic liver profiles

**Fig 2:**
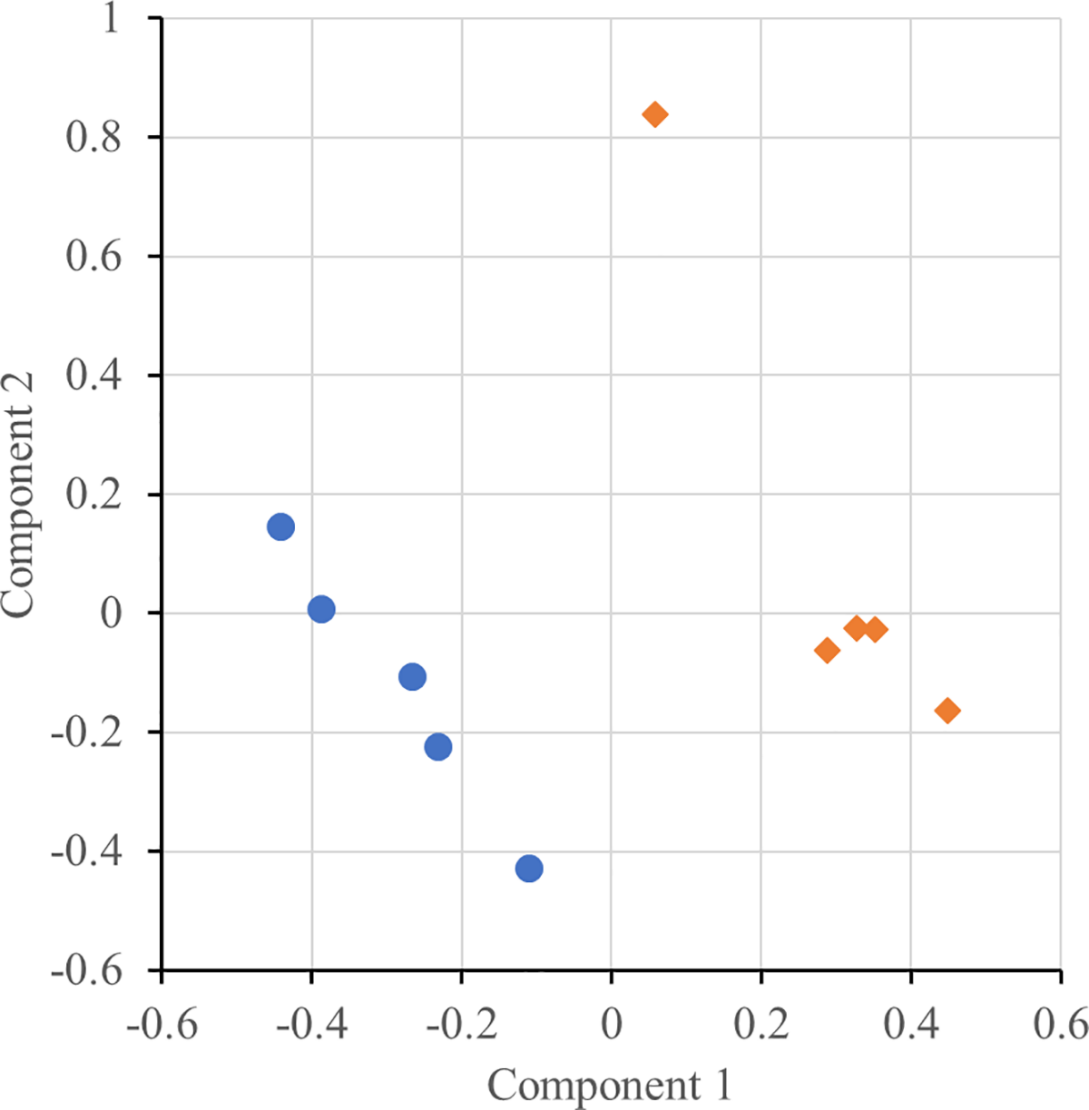
Scatter plot of the first two sPLS-DA components obtained from the liver proteome of five Italian Duroc pigs (blue circles) and five Italian Large White pigs (orange diamonds).

Differences at the proteome level between Italian Duroc and Italian Large White pigs were investigated by using the multivariate approach of sPLS-DA. This technique was coupled with a statistical procedure aimed at evaluating the stability and significance of the proteins selected as differentially expressed. The scatter plot of the first two sPLS-DA components shows that animals of the same breed clustered together, indicating that the identified proteins can discriminate animals of these two pig breeds (Fig 2). According to the stability test, 33 proteins (16.5% on the total) had a *P* ≤ 0.10 (defined as a threshold of significance, based on the validation procedure; [18]). The sPLS-DA regression coefficient of 57 proteins (28.5%) was equal to 0, indicating that these proteins do not have any weight in the classification derived by the breed. At the significance test, 56 out of 57 proteins had a *P* ≤ 0.10. A total of 25 out of 200 identified proteins (12.5%) were both stable (in terms of statistically assigned condition) and significantly differentially expressed (*P* ≤ 0.10) (Table 1). Among these proteins, 14 (56%) showed an increased abundance in Italian Duroc pigs, while the remaining 11 (44%) had a higher quantification level in Italian Large White pigs. Details about proteins with significant different quantification between the two breeds are presented in Table 1. Four of these proteins (two with the highest expression in Italian Duroc pigs: 3-ketoacyl-CoA thiolase, mitochondrial (ACAA2) and histone H2B type 2-F (HIST2H2BF); and two with the highest expression in Italian Large White pigs: ketohexokinase (KHK) and carboxylesterase 3 (CES3) showing one of the two calculated *P* values ≤0.01 and the other with ≤0.05, were considered the top proteins identified in this study differentiating the two analysed breeds.

**Table 1:** Differentially abundant proteins identified from label-free mass spectrometry analysis of liver samples of Italian Duroc (ID) and Italian Large White (ILW) pigs. Proteins are ordered according to their fold change score.

UniProtKB^1^	Gene Name	Protein	Peptides	PA_IDU_^2^	PA_ILW_^3^	FC^4^	Direction^5^	*P*_st_^6^	*P*_si_^7^
P51781	GSTA1	Glutathione S-transferase A1	5	-0.41	0.54	0.52	ILW	0.077	0.026
I3LVE1	KHK	Ketohexokinase	3	-0.24	0.64	0.54	ILW	0.005	0.027
I3LEI5	CES3	Carboxylesterase 3	2	-0.64	0.23	0.55	ILW	0.006	0.014
B1A8Z3	PYGL	Glycogen phosphorylase, liver form	5	-0.45	0.38	0.56	ILW	0.022	0.036
Q9TV69	DHDH (SUS2DD)	Trans-1,2-dihydrobenzene-1,2-diol dehydrogenase	3	-0.39	0.30	0.62	ILW	0.091	0.016
A0A286ZKH3	SPR	Sepiapterin reductase	5	-0.41	0.19	0.66	ILW	0.042	0.044
A0A286ZIW5	ILVBL	Acetolactate synthase-like protein	2	-0.21	0.34	0.68	ILW	0.031	0.027
F1RII7	HBB	Hemoglobin subunit beta	8	-0.20	0.33	0.69	ILW	0.080	0.042
F1RQP0	PRDX5	Peroxiredoxin-5, mitochondrial	8	-0.20	0.31	0.70	ILW	0.042	0.024
F1SLX5	AASS	Alpha-aminoadipic semialdehyde synthase, mitochondrial	2	-0.42	-0.02	0.76	ILW	0.073	0.029
A5YV76	FASN	Fatty acid synthase	7	-0.12	0.17	0.82	ILW	0.086	0.035
F1SSS0	CPS1	Carbamoyl-phosphate synthase [ammonia], mitochondrial	39	0.12	-0.07	1.14	IDU	0.084	0.066
F1RKG8	PEBP1	Phosphatidylethanolamine-binding protein 1	7	0.21	-0.24	1.37	IDU	0.073	0.038
D0G0B3	ACAA2	3-ketoacyl-CoA thiolase, mitochondrial	10	0.36	-0.20	1.48	IDU	0.010	0.016
F1S2X3	ECHDC1	Ethylmalonyl-CoA decarboxylase	2	0.09	-0.49	1.50	IDU	0.086	0.041
F1S0C1	ADH5	Alcohol dehydrogenase class-3	4	0.38	-0.24	1.53	IDU	0.068	0.052
F1RMH5	UROC1	Urocanate hydratase	3	0.25	-0.41	1.58	IDU	0.029	0.035
I3LP02	ACAT1	Acetyl-CoA acetyltransferase, mitochondrial	2	0.22	-0.57	1.73	IDU	0.099	0.023
F1RL81	HSD17B14	17-beta-hydroxysteroid dehydrogenase 14	4	0.29	-0.51	1.74	IDU	0.047	0.026
A0A287AR55	FH	Fumarate hydratase, mitochondrial	3	0.40	-0.43	1.78	IDU	0.059	0.044
F1RWY0	RGN	Regucalcin	9	0.40	-0.45	1.80	IDU	0.051	0.055
F2Z558	YWHAZ	14-3-3 protein zeta/delta	2	0.33	-0.64	1.96	IDU	0.082	0.048
UPI0002105648	HIST2H2BF	Histone H2B type 2-F	2	0.58	-0.40	1.97	IDU	0.009	0.008
F1RIF3	FAH	Fumarylacetoacetase	3	0.54	-0.48	2.02	IDU	0.005	0.052
I3LDY2	LOC100625049	Uncharacterized protein	3	0.54	-0.85	2.63	IDU	0.012	0.020

^1^UniProtKB accession number as derived from the Pig PeptideAtlas resource

^2^Average abundance level of protein in Italian Duroc pigs

^3^Average abundance level of protein in Italian Large White pigs

^4^Fold change

^5^“ID” indicates protein abundance higher in Italian Duroc pigs than Italian Large White pigs while ‘ILW’ indicates protein abundance higher in Italian Large White pigs than Italian Duroc pigs

^6^*p*-value at the stability test

^7^*p*-value at the significance test.

### Functional inference of proteomic differences between breeds

**Fig 3:**
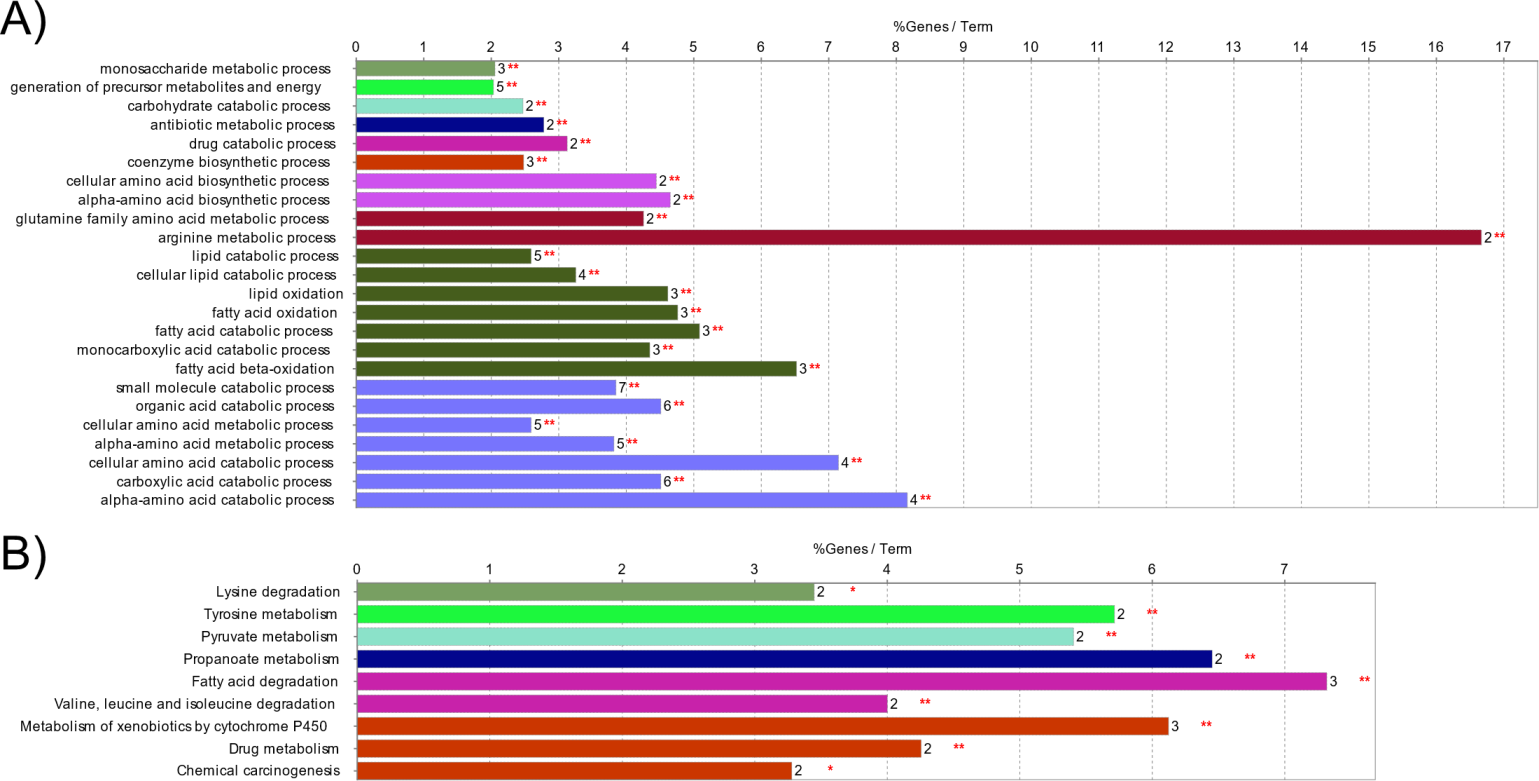
Functional analysis of the 25 differentially abundant proteins between Italian Duroc and Italian Large White pigs. The two panels show the result of gene enrichment analyses over the A) Gene Ontology–Biological Process branch, and B) over the KEGG pathway database. Bars represent the percentage of input proteins found associated with respect to the number of proteins directly annotated with the functional term. The number of input proteins related to the term and the term significance are reported next to each bar. Detailed statistics are reported in Table 2 and Table 3, respectively. In each panel, bars sharing a specific color are clustered in the same functional group (see Table 2).

Biological processes and pathways involving the 25 differentially abundant proteins were investigated through functional association analysis. Enrichment analyses were carried out in Cytoscape (ClueGO package). Analyses were run over the GO:BP and KEGG pathway databases, separately. Two proteins (HIST2H2BF and LOC100625049) were not in the ClueGO annotation sets. A total of 24 GO:BP terms (involving 14 differentially abundant proteins) were retrieved (Table 2, Fig 3A). These biological processes can be summarized in the following functional groups (G, in Table 2): (i) metabolism of carbohydrates and energy (G1—G3), (ii) metabolism of antibiotics/drugs (G4—G5), (iii) metabolism of cofactors (G6), (iv) metabolism of amino-acids (G7—G8; G10), (v) metabolism of lipids (G9—G10). It is interesting to note that processes related to the glutamine family amino acids and lipids involve proteins with a higher abundance in Italian Duroc than in Italian Large White pigs. Processes related to organic acid catabolism cellular/alpha amino acid metabolism showed the same direction, except for the aminoadipate-semialdehyde synthase (AASS) protein, that was more expressed in the Italian Large White pigs. This inversion was also evident for proteins involved in the carbohydrate catabolic process that showed a higher abundance in Italian Large White than in Italian Duroc pigs.

**Table 2:** Over-represented biological processes (GO:BP) associated to up or down regulated proteins in the breed comparison.

Term	Description	Functional group^1^	p-value^2^	% of associated proteins^3^	N. of proteins	Up or down regulated proteins ^4^
GO:0005996	monosaccharide metabolic process	G1	2.26E-03	2.05	3	KHK↓, RGN↑, SUS2DD↓
GO:0006091	generation of precursor metabolites and energy	G2	1.23E-04	2.03	5	AASS↓, ACAT1↑, ADH5↑, FH↑, PYGL↓
GO:0016052	carbohydrate catabolic process	G3	7.63E-03	2.47	2	PYGL↓, SUS2DD↓
GO:0016999	antibiotic metabolic process	G4	6.36E-03	2.78	2	ADH5↑, FH↑
GO:0042737	drug catabolic process	G5	5.31E-03	3.13	2	AASS↓, ACAT1↑
GO:0009108	coenzyme biosynthetic process	G6	1.40E-03	2.48	3	ACAT1↑, RGN↓, SPR↓
GO:0008652	cellular amino acid biosynthetic process	G7	2.95E-03	4.44	2	AASS↓, CPS1↑
GO:1901607	alpha-amino acid biosynthetic process	G7	2.86E-03	4.65	2	AASS↓, CPS1↑
GO:0009064	glutamine family amino acid metabolic process	G7	3.05E-03	4.26	2	CPS1↑, FAH↑
GO:0006525	arginine metabolic process	G8	2.81E-04	16.67	2	CPS1↑, FAH↑
GO:0016042	lipid catabolic process	G9	4.45E-05	2.59	5	ACAA2↑, ACAT1↑, CPS1↑, ECHDC1↑, HSD17B141↑
GO:0044242	cellular lipid catabolic process	G9	1.16E-04	3.25	4	ACAA2↑, ACAT1↑, CPS1↑, ECHDC1↑
GO:0034440	lipid oxidation	G9	2.82E-04	4.62	3	ACAA2↑, ACAT1↑, ECHDC1↑
GO:0019395	fatty acid oxidation	G9	2.81E-04	4.76	3	ACAA2↑, ACAT1↑, ECHDC1↑
GO:0009062	fatty acid catabolic process	G9	2.54E-04	5.08	3	ACAA2↑, ACAT1↑, ECHDC1↑
GO:0072329	monocarboxylic acid catabolic process	G9	2.89E-04	4.35	3	ACAA2↑, ACAT1↑, ECHDC1↑
GO:0006635	fatty acid beta-oxidation	G9	1.33E-04	6.52	3	ACAA2↑, ACAT1↑, ECHDC1↑
GO:0044282	small molecule catabolic process	G10	2.21E-07	3.85	7	AASS↓, ACAA2↑, ACAT1↑, ECHDC1↑, FAH↑, SUS2DD↓, UROC1↑
GO:0016054	organic acid catabolic process	G10	5.77E-07	4.51	6	AASS↓, ACAA2↑, ACAT1↑, ECHDC1↑, FAH↑, UROC1↑
GO:0006520	cellular amino acid metabolic process	G10	4.45E-05	2.59	5	AASS↓, ACAT1↑, CPS1↑, FAH↑, UROC1↑
GO:1901605	alpha-amino acid metabolic process	G10	1.00E-05	3.82	5	AASS↓, ACAT1↑, CPS1↑, FAH↑, UROC1↑
GO:0009063	cellular amino acid catabolic process	G10	8.06E-06	7.14	4	AASS↓, ACAT1↑, FAH↑, UROC1↑
GO:0046395	carboxylic acid catabolic process	G10	5.77E-07	4.51	6	AASS↓, ACAA2↑, ACAT1↑, ECHDC1↑, FAH↑, UROC1↑
GO:1901606	alpha-amino acid catabolic process	G10	7.81E-06	8.16	4	AASS↓, ACAT1↑, FAH↑, UROC1↑

^1^Groups of closely related terms

^2^Benjamini-Hochberg corrected *p*-values

^3^Percentage of input proteins found associated with respect to the number of proteins directly annotated with the functional term

^4^The symbol ↑ indicates protein abundance higher in Italian Duroc pigs than Italian Large White pigs while the symbol ↓ indicates protein abundance higher in Italian Large White pigs than Italian Duroc pigs

Over-representation analysis over the KEGG pathway database highlighted a total of nine pathways (Table 3, Fig 3B) related to the metabolism of lipids, amino-acids, carbohydrates and chemicals (as previously showed from the analysis over the GO:BP database). Nine proteins were involved in these pathways. Again, in this case, all the differentially abundant proteins involved in the fatty acid catabolism had higher concentration in Italian Duroc pigs than in Italian Large White pigs. Moreover, in addition to the glutamine family amino acids, the pathways involving valine, leucine, isoleucine and tyrosine showed the same behaviour.

**Table 3:** Over-represented KEGG pathways associated to up or down regulated proteins in the breed comparison.

Term	Description	p-value^1^	% of associated proteins^2^	N. of proteins	Up or down regulated proteins^3^
KEGG:00310	Lysine degradation	1.06E-02	3.45	2	AASS↓, ACAT1↑
KEGG:00350	Tyrosine metabolism	7.88E-03	5.71	2	ADH5↑, FAH↑
KEGG:00620	Pyruvate metabolism	7.04E-03	5.41	2	ACAT1↑, FH↑
KEGG:00640	Propanoate metabolism	8.27E-03	6.45	2	ACAT1↑, ECHDC1↑
KEGG:00071	Fatty acid degradation	1.28E-03	7.32	3	ACAA2↑, ACAT1↑, ADH5↑
KEGG:00280	Valine, leucine and isoleucine degradation	9.07E-03	4.00	2	ACAA2↑, ACAT1↑
KEGG:00980	Metabolism of xenobiotics by cytochrome P450	1.09E-03	6.12	3	ADH5↑, GSTA1↓, SUS2DD↓
KEGG:00982	Drug metabolism	9.38E-03	4.26	2	ADH5↑, GSTA1↓
KEGG:05204	Chemical carcinogenesis	1.04E-02	3.28	2	ADH5↑, GSTA1↓

^1^Benjamini-Hochberg corrected *p*-values

^2^Percentage of input proteins found associated with respect to the number of proteins directly annotated with the functional term

^3^The symbol ↑ indicates protein abundance higher in Italian Duroc pigs than Italian Large White pigs while the symbol ↓ indicates protein abundance higher in Italian Large White pigs than Italian Duroc pigs.

**Fig 4:**
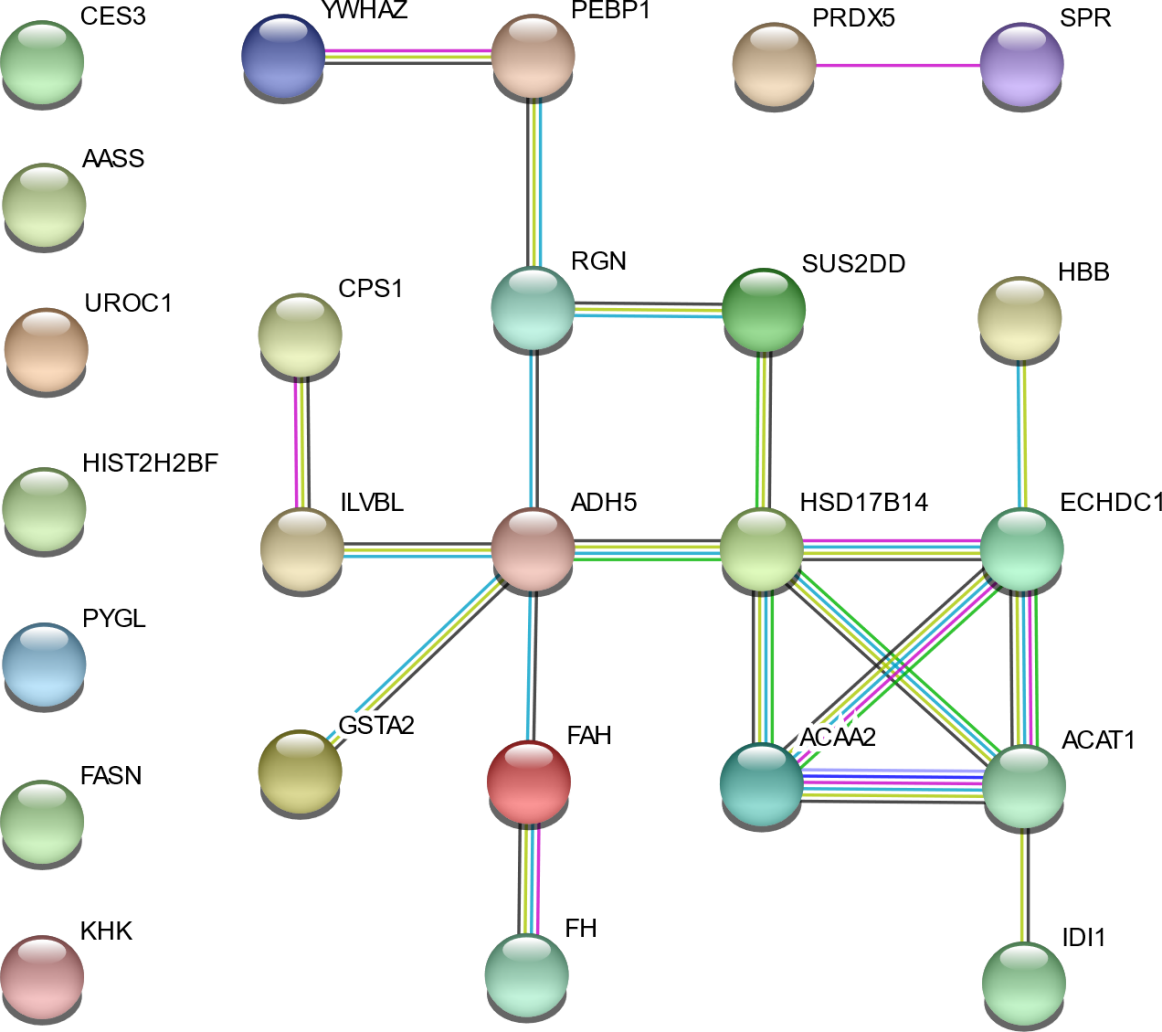
Protein-protein interaction map of the 25 differentially abundant proteins. Interactions are based on STRING v.10.5 and are shown in different colors: cyan is from curated databases, magenta is experimentally determined, dark green is gene neighborhood, blue is gene co-occurrence, light green is textmining, black is co-expression and purple is protein homology.

STRING was used to highlight the functional connections established among the differentially abundant proteins. The analysis revealed a connected protein network (Fig 4) divided in: (i) one big module composed by 16 nodes (64%) and 19 links, (ii) a small component of two proteins (8%) and (iii) seven singletons (28%). The resulting network showed a PPI enrichment *p*-value of 3.67×10^−10^ (three expected edges vs. 20 detected edges) indicating that proteins are at least partially biologically connected. In this network most of the proteins interacted with only one or two other partners (average node degree equal to 1.6). However, two proteins (ADH5 and HSD17B14; with higher expression in Italian Duroc than in Italian Large White pigs) presented the highest degree of connection (six edges), which may assign to them a role as “hub” proteins playing a putative function of controllers inside biochemical pathways that could potentially lead to cascade of protein expression differences. In addition, the big module clustered all proteins that were included in the GO and KEGG enriched processes clearly differentiating (in terms of direction of the relative level of expression) Italian Duroc and Italian Large White liver proteomic profiles.

S3 Table reports information on the potential links between differentially expressed proteins identified in the comparison between breeds and QTL mapped in the corresponding gene regions, as retrieved from the pigQTL database. A total of 12 out of 25 up or down regulated proteins identified in the breed comparative analysis resulted located in ± 50 kbp surrounding 23 QTL regions (for a total of 40 protein/QTL combinations), that might be directly or indirectly (in a broad sense) affected by the protein functions. Six of these proteins were up-regulated in Italian Duroc pigs (ECHDC1, HSD17B14, FAH, UROC1, ACAT1, CPS1) and six in Italian Large White pigs (PRDX5, SPR, GSTA1, FASN, KHK, CES3).

## Discussion

The pig is one of the most important animal species used as protein source for human consumption that is also considered a suitable model for several biomedical aspects related to liver functions [41, 42]. In spite of that, thus far only few studies have investigated the pig liver proteome [9, 11–13, 43–52]. Most of these liver proteomic investigations compared proteomic profiles of pigs under different experimental conditions or treatments or just wanted to extend the porcine liver proteome map. Our work not only contributed to obtain a more detailed picture of the protein expressed in pig liver but also provided a first comparative analysis of the liver proteome of two important heavy pig breeds, Italian Duroc and Italian Large White. These breeds are used in crossbreeding programmes to obtain commercial slaughtered pigs, maximizing the effect of heterosis. Italian Large White pigs are mainly selected for meat quality and carcass traits and to maximize maternal reproduction efficiency. Italian Duroc pigs are mainly used as terminal sires and are selected to maximize growth, feed efficiency and improve meat quality parameters [21]. As the liver can be considered a key organ for translating growth rate, feed efficiency and performance potentials of the animals by exploiting tissue specific metabolic functions, the evaluation of proteome differences between Italian Duroc and Italian Large White pigs could highlight biological aspects (that, in turn, are derived by genetic factors) that distinguish and characterize these breeds and that might determine their peculiar production potentials.

It is clear that any proteomic study cannot disclose in a single analysis or method all proteins that construct a tissue or an organ for intrinsic limits of the available technologies and difficulties to discriminate and separate all proteins present in complex matrices. The predicted human proteome has been estimated to be constituted by at least 20,000 proteins (without considering splicing variants), about 59% of which might be expressed in the liver [53]; a similar fraction could be expected in the porcine liver. Therefore, the liver could be considered one of the richest organs in terms of number of proteins. However, studies in pig liver reported, in most cases, tens or just a few hundreds of proteins. For example, Caperna *et al*. [11] using 2DE produced two liver porcine proteomic maps, one from the cytosol fraction and another one from the membrane fraction. A total of 728 proteins spots were picked and analysed by MS resulting in a total of 282 unique identified proteins. Golovan *et al*. [44] using isobaric tag for relative and absolute quantification (iTRAQ) proteomics identified a higher number of proteins (i.e. n. = 880) in the liver proteomic profiles obtained from two pig lines (transgenic Enviropig and conventional Yorkshire).

In our study, the label‐free LC‐MS analysis revealed a total of 501 different proteins that, after stringent filtering were identified as 329 unique proteins, of which 200 were then considered in the comparison between the two breeds (S1 and S2 Tables). Many of the identified proteins (e.g. peroxiredoxin, acetyl-CoA, carboxylesterase, fumarylacetoacetase) were also identified in other studies carried out in pigs or other livestock species [11, 44, 54]. Most of the identified proteins were involved in several metabolic processes that characterize the liver functions (Fig 1). Differences among studies might be due to different methods of protein extraction employed and by the peptide separation and identification technologies. Different approaches and methods have different efficiencies and throughput and resolution potentials which determine, in turn, the number of detected proteins. For example, the 2DE study of Caperna *et al*. [11] identified a total of 282 unique porcine liver proteins, the majority of which were from the cytosol fractions. It is well know that this method is technically challenging and is not optimized for the analysis of very hydrophobic and/or membrane proteins [55]. Using iTRAQ, Golovan *et al*. [44] identified a higher range of proteins in the pig proteome, but only few of them (i.e. two and four) were differentially expressed between breeds and sexes, respectively. In this label-based method, stable isotopes are employed to create specific mass tags that can be recognized by the mass spectrometer and can provide, at the same time, the basis for the quantification. One of the main advantage of this method is that it allows, in a single MS run, the simultaneous analysis of many samples, reducing analytical variability [56]. On the other hand, the label-free method that we applied provides a higher dynamic range of quantification than that obtained by stable isotope labelling methods [57].

To our knowledge, this work is the first study which applies a label-free method to unravel the pig liver proteome. In the label-free study of Miller *et al*. [54], that compared the liver proteome of two sheep breeds, a larger number of proteins was identified (2,445 vs 501 of our work), probably due to differences in sensitivity of the used instruments. Miller *et al*. [54] and our work had a similar objective, i.e. identify proteomic differences between breeds. In both studies that were based on a similar experimental design (both included comparative analyses between breeds), the same percentage of proteins (about 8%) were declared as differentially expressed between the two analysed genetic types (within species). Even if it could be quite speculative, this general overlapping gross picture might define similar levels of underlying genetic diversity between the compared groups of the two species.

sPLS-DA plot showed distinct clustering, which clearly segregated the samples of the two analysed pig breeds based on their liver proteomic profiles (Fig 2). This pattern is in agreement with our previous results obtained by analyzing targeted metabolomic profiles of plasma and serum of Italian Duroc and Italian Large White pigs [18]. Liver interplays with blood exchanging the products of their respective anabolism and catabolism and many other components, depicting a metabolic picture that could be observed at the organ or tissue level as well in the circulating tissue and in its fractions (i.e. plasma and serum).

Among the 25 proteins that were up- or down-regulated between the two pig breeds, the top differentially expressed proteins (ACAA2, HIST2H2BF, CES3 and KHK) according to their functions, may contribute to explain in part the phenotypic differences between Italian Duroc and Italian Large White animals, complementing the metabolomic profile differences already reported in a previous study [18]. Carboxylesterase 3 (CES3) was up-regulated in Italian Large White. This is a member of enzymes of the endoplasmic reticulum that hydrolyses a variety of esters, carbamates, amides and similar structures of drugs and xenobiotics [58]. This enzyme is involved in hepatic very low-density lipoprotein (VLDL) assembly and in basal lipolysis of adipose tissues. Ablation of carboxylesterase 3 expression in mice results in reduced circulating plasma triacylglycerol, apolipoprotein B, and fatty acid levels and increased food intake and energy expenditure [59, 60]. Moreover, the porcine *CES3* gene is located on porcine chromosome (SSC) 6 in a QTL region for residual feed intake (that might be related to feed intake and metabolism efficiency; S3 Table) [40]. Differences of hepatic CES3 levels between Italian Large White and Italian Duroc might be in line with a higher feed efficiency of the latter breed. ACAA2, among the top up-regulated proteins in Italian Duroc pigs, is a mitochondrial enzyme catalysing the last step in fatty acid oxidation which releases acetyl CoA for the Krebs cycle. For its role, it is considered one of the key enzymes in lipid metabolism [61], further evidencing differences related to fat related pathways. Again, ACAT1, that is involved in cellular cholesterol homeostasis, macrophage cholesterol metabolism, isoleucine metabolism and ketogenesis (i.e. Hai *et al*. [62]), was up-regulated in Italian Duroc pigs. The *ACAT1* gene is located on SSC9 in a region in which several QTLs for average daily gain and intramuscular fat content have been identified (S3 Table). In addition, this protein has been already identified to be differentially expressed in a proteomic analysis of skeletal muscles between pigs with extreme values of intramuscular fat content [63], strengthening a role of lipid metabolism differences between pig breeds that could result in different phenotypic traits [23, 64]. Moreover, a few other hepatic differences between Italian Duroc and Italian Large White pigs highlighted also metabolic differences in fatty acid metabolism. The *ECHDC1* gene, which encodes for a protein involved in the mitochondrial fatty acid oxidation, is located in a region of the SSC1 overlapping a QTL region involved in the saturated fatty acid content (S3 Table). FASN (up-regulated in Italian Large White pigs) is an enzyme that catalyses the biosynthesis of palmitic acid. The porcine *FASN* gene is located on SSC12 in a region in which two QTLs affecting the myristic and palmitic acids have been mapped and for which *FASN* was pointed out as the most plausible candidate gene [65]. Histone H2B (HIST1H2BA), is a member of the histone family, which are basic nuclear proteins that are responsible for the nucleosome structure of the chromosomal fiber in eukaryotes [66]. This family of proteins and peptides derived from them were found to be part of the antimicrobial defense of many species [67–69]. For example, Li et al [68] highlighted the antimicrobial activity of HIST1H2BA and other histone proteins in the chicken liver extract. In our study, HIST2H2BF was up-regulated in the Italian Duroc breed, which may allude to a stronger resistance to disease of this breed, that is, in general considered the most rustic breed among all commercial pig breeds [70].

These specific differences highlighted by the function of the proteins mentioned above (and all other proteins that contributed to produce “breed specific” proteomic profiles; Table 1) can be summarized in few biological processes that could be identified summing up their roles, matching again some of the metabolomic differences (described with biogenic amines and sphingomyelins) that we already reported between these two breeds [18]. Protein differentially expressed were widely distributed among the known biological processes associated with liver metabolism such as metabolism of lipids, metabolism of amino-acids, metabolism of carbohydrates, metabolism of cofactors and metabolism of antibiotics/drugs. Liver proteomic profiles we obtained for Italian Duroc and Italian Large White pigs seems to match production characteristics of these breeds that have been developed over decades of divergent directional selection originally determined by the genetic pools constituting these two heavy pig breeds.

## Conclusions

In this study we reported results from the first proteomic investigation of the liver of heavy pigs using a label-free proteomic approach. Despite this work should be considered a pilot study and proteomic differences might be confirmed with other approaches, the 25 identified proteins up- or down-regulated in the compared breeds were able to discriminate the liver proteomic profile of pigs belonging to the Italian Duroc and Italian Large White breeds. These results indirectly demonstrated that breed differences (underlying general genetic differences) can be highlighted at the liver proteome level. These different proteomic profiles could be useful to describe breed-specific metabolic characteristics. We also provided evidences that quantitative proteomic approaches are useful to describe internal phenotypes that could be important to link and dissect external (production) and complex traits. The obtained proteomic differences highlighted several biomarkers that can be potentially useful to define new molecular phenotypes for novel applications in pig breeding programmes.

## Supporting information

S1 Table List of the 329 proteins used to group the liver proteins according to their different biological processes. (XLSX)

S2 Table Full list of the 200 proteins analysed in this study. (XLSX)

S3 Table Links between differentially expressed proteins identified in the comparison between breeds and QTLs mapped in the corresponding gene regions (± 50 kbp). (DOCX)

## References

[1] Trefts E, Gannon M, Wasserman DH. The liver. Current biology: CB. 2017;27(21):R1147–R1151. 10.1016/j.cub.2017.09.019 29112863 PMC5897118

[2] Rui L. Energy Metabolism in the Liver. Comprehensive Physiology. 2014;4(1):177–197. 10.1002/cphy.c130024 24692138 PMC4050641

[3] Foster LJ, de Hoog CL, Zhang Y, Zhang Y, Xie X, Mootha VK, et al A Mammalian Organelle Map by Protein Correlation Profiling. Cell. 2006;125(1):187–199. 10.1016/j.cell.2006.03.022 16615899

[4] Ying W, Jiang Y, Guo L, Hao Y, Zhang Y, Wu S, et al A dataset of human fetal liver proteome identified by subcellular fractionation and multiple protein separation and identification technology. Mol Cell Proteomics. 2006;5(9):1703–1707. 10.1074/mcp.M500344-MCP200 16815949

[5] Zheng J, Gao X, Mato J, Beretta L, He F. Report of the 9th HLPP Workshop October 2007, Seoul, Korea. Proteomics. 2008;8(17):3420–3423. 10.1002/pmic.200800432 18683817 PMC4601560

[6] Dawson HD, Chen C, Gaynor B, Shao J, Urban JF Jr. The porcine translational research database: a manually curated, genomics and proteomics-based research resource. BMC Genomics. 2017;18(1):643 Epub 2017/08/24. 10.1186/s12864-017-4009-7 28830355 PMC5568366

[7] Marx H, Hahne H, Ulbrich SE, Schnieke A, Rottmann O, Frishman D, et al Annotation of the Domestic Pig Genome by Quantitative Proteogenomics. J Proteome Res. 2017;16(8):2887–2898. 10.1021/acs.jproteome.7b00184 28625053

[8] Bassols A, Costa C, Eckersall PD, Osada J, Sabria J, Tibau J. The pig as an animal model for human pathologies: A proteomics perspective. Proteomics Clin Appl. 2014;8(9–10):715–731. 10.1002/prca.201300099 25092613

[9] Cui Y, Hao Y, Li J, Bao W, Li G, Gao Y, et al Chronic Heat Stress Induces Immune Response, Oxidative Stress Response, and Apoptosis of Finishing Pig Liver: A Proteomic Approach. International Journal of Molecular Sciences. 2016;17(5):393. 10.3390/ijms17050393 PMC4881434 27187351

[10] Timperio AM, D'Alessandro A, Pariset L, D'Amici GM, Valentini A, Zolla L. Comparative proteomics and transcriptomics analyses of livers from two different Bos taurus breeds: “Chianina and Holstein Friesian”. Journal of Proteomics. 2009; 73(2):309–322. 10.1016/j.jprot.2009.09.015 19782776

[11] Caperna TJ, Shannon AE, Garrett WM. A gel-based reference map of the porcine hepatocyte proteome. Domest Anim Endocrinol. 2008;35(2):142–156. 10.1016/j.domaniend.2007.12.004 18538972

[12] Wang J, Chen L, Li D, Yin Y, Wang X, Li P, et al Intrauterine growth restriction affects the proteomes of the small intestine, liver, and skeletal muscle in newborn pigs. J Nutr. 2008;138(1):60–66. 10.1093/jn/138.1.60 18156405

[13] Liu C, Lin G, Wang X, Wang T, Wu G, Li D, et al Intrauterine growth restriction alters the hepatic proteome in fetal pigs. J Nutr Biochem. 2013;24(6):954–959. 10.1016/j.jnutbio.2012.06.016 22959055

[14] Neilson KA, Ali NA, Muralidharan S, Mirzaei M, Mariani M, Assadourian G, et al Less label, more free: approaches in label-free quantitative mass spectrometry. Proteomics. 2011;11(4):535–53. 10.1002/pmic.201000553 21243637

[15] Tang X, Meng Q, Gao J, Zhang S, Zhang H, Zhang M. Label-free Quantitative Analysis of Changes in Broiler Liver Proteins under Heat Stress using SWATH-MS Technology. Scientific Reports. 2015;5:15119 10.1038/srep15119 26459884 PMC4602270

[16] Fontanesi L. Merging Metabolomics, Genetics, and Genomics in Livestock to Dissect Complex Production Traits In: Kadarmideen HN, editor. Systems Biology in Animal Production and Health, Vol 1 Cham: Springer International Publishing; 2016 p. 43–62.

[17] Fontanesi L. Metabolomics and livestock genomics: Insights into a phenotypin frontier and its applications in animal breeding. *Animal Frontiers*. 2016;6(1):73–79.

[18] Bovo S, Mazzoni G, Galimberti G, Calò DG, Fanelli F, Mezzullo M, et al Metabolomics evidences plasma and serum biomarkers differentiating two heavy pig breeds. Animal. 2016;10(10):1741–1748. 10.1017/S1751731116000483 27055632

[19] Cilla I, Altarriba J, Guerrero L, Gispert M, Martínez L, Moreno C, et al Effect of different Duroc line sires on carcass composition, meat quality and dry cured ham acceptability. Meat Science. 2006;72(2):252 10.1016/j.meatsci.2005.07.010 22061552

[20] Edwards SA, Wood JD, Moncrieff CB, Porter SJ. Comparison of the Duroc and Large White as terminal sire breeds and their effect on pigmeat quality. Animal Science. 1992;54(2):289–297.

[21] Bosi P, Russo V. The production of the heavy pig for high quality processed products. Italian Journal of Animal Science. 2004;3(4):309–321.

[22] Fontanesi L, Galimberti G, Calo DG, Fronza R, Martelli PL, Scotti E, et al Identification and association analysis of several hundred single nucleotide polymorphisms within candidate genes for back fat thickness in Italian Large White pigs using a selective genotyping approach. J Anim Sci. 2012;90(8):2450–2464. 10.2527/jas.2011-4797 22367074

[23] Bertolini F, Schiavo G, Galimberti G, Bovo S, D'Andrea M, Gallo M, et al Genome-wide association studies for seven production traits highlight genomic regions useful to dissect dry-cured ham quality and production traits in Duroc heavy pigs. Animal. 2018:1–8. 10.1017/S1751731118000757 29706143

[24] Bovo S, Schiavo G, Mazzoni G, Dall'Olio S, Galimberti G, Calò DG, et al Genome‐wide association study for the level of serum electrolytes in Italian Large White pigs. Animal Genetics. 2016;47(5):597–602. 10.1111/age.12459 27296164

[25] Wisniewski JR, Zougman A, Nagaraj N, Mann M. Universal sample preparation method for proteome analysis. Nat Meth. 2009;6(5):359–362. 10.1038/nmeth.1322 19377485

[26] Hesselager MO, Codrea MC, Sun Z, Deutsch EW, Bennike TB, Stensballe A, et al The Pig PeptideAtlas: A resource for systems biology in animal production and biomedicine. Proteomics. 2016;16(4):634–644. 10.1002/pmic.201500195 26699206 PMC4786621

[27] Deutsch EW, Mendoza L, Shteynberg D, Slagel J, Sun Z, Moritz RL. Trans-Proteomic Pipeline, a standardized data processing pipeline for large-scale reproducible proteomics informatics. Proteomics Clin Appl. 2015;9(7–8):745–754. 10.1002/prca.201400164 25631240 PMC4506239

[28] Keller A, Nesvizhskii AI, Kolker E, Aebersold R. Empirical statistical model to estimate the accuracy of peptide identifications made by MS/MS and database search. Anal Chem. 2002;74(20):5383–5392. 12403597 10.1021/ac025747h

[29] Nesvizhskii AI, Keller A, Kolker E, Aebersold R. A statistical model for identifying proteins by tandem mass spectrometry. Anal Chem. 2003;75(17):4646–4658. 14632076 10.1021/ac0341261

[30] MacLean B, Tomazela DM, Shulman N, Chambers M, Finney GL, Frewen B, et al Skyline: an open source document editor for creating and analyzing targeted proteomics experiments. Bioinformatics. 2010;26(7):966–968. 10.1093/bioinformatics/btq054 20147306 PMC2844992

[31] Polpitiya AD, Qian WJ, Jaitly N, Petyuk VA, Adkins JN, Camp DG 2nd, et al DAnTE: a statistical tool for quantitative analysis of -omics data. Bioinformatics. 2008;24(13):1556–8. 10.1093/bioinformatics/btn217 18453552 PMC2692489

[32] Vizcaino JA, Csordas A, del-Toro N, Dianes JA, Griss J, Lavidas I, et al 2016 update of the PRIDE database and its related tools. Nucleic Acids Res. 2016;44(D1):D447–D456. 10.1093/nar/gkv1145 26527722 PMC4702828

[33] Chun H, Keleş S. Sparse partial least squares regression for simultaneous dimension reduction and variable selection. Journal of the Royal Statistical Society Series B, Statistical Methodology. 2010;72(1):3–25. 10.1111/j.1467-9868.2009.00723.x 20107611 PMC2810828

[34] Bovo S, Mazzoni G, Calo DG, Galimberti G, Fanelli F, Mezzullo M, et al Deconstructing the pig sex metabolome: Targeted metabolomics in heavy pigs revealed sexual dimorphisms in plasma biomarkers and metabolic pathways. J Anim Sci. 2015;93(12):5681–5693. 10.2527/jas.2015-9528 26641177

[35] Le Cao KA, Boitard S, Besse P. Sparse PLS discriminant analysis: biologically relevant feature selection and graphical displays for multiclass problems. BMC Bioinformatics. 2011;12:253 Epub 2011/06/23. 10.1186/1471-2105-12-253 21693065 PMC3133555

[36] R Core Team. R: A language and environment for statistical computing. Vienna, Austria 2015. URL http://www.R-project.org/.

[37] Shannon P, Markiel A, Ozier O, Baliga NS, Wang JT, Ramage D, et al Cytoscape: a software environment for integrated models of biomolecular interaction networks. Genome Res. 2003;13(11):2498–2504. 10.1101/gr.1239303 14597658 PMC403769

[38] Bindea G, Mlecnik B, Hackl H, Charoentong P, Tosolini M, Kirilovsky A, et al ClueGO: a Cytoscape plug-in to decipher functionally grouped gene ontology and pathway annotation networks. Bioinformatics. 2009;25(8):1091–1093. 10.1093/bioinformatics/btp101 19237447 PMC2666812

[39] Szklarczyk D, Morris JH, Cook H, Kuhn M, Wyder S, Simonovic M, et al The STRING database in 2017: quality-controlled protein-protein association networks, made broadly accessible. Nucleic Acids Res. 2017;45(D1):D362–D368. 10.1093/nar/gkw937 27924014 PMC5210637

[40] Hu Z-L, Park CA, Reecy JM. Developmental progress and current status of the Animal QTLdb. Nucleic Acids Research. 2016;44(Database issue):D827–D833. 10.1093/nar/gkv1233 26602686 PMC4702873

[41] de Almeida AM, Bendixen E. Pig proteomics: A review of a species in the crossroad between biomedical and food sciences. Journal of Proteomics. 2012;75(14):4296–4314. 10.1016/j.jprot.2012.04.010 22543283

[42] Lunney JK. Advances in swine biomedical model genomics. Int J Biol Sci. 2007;3 10.7150/ijbs.3.179 PMC1802015 17384736

[43] Junghans P, Kaehne T, Beyer M, Metges CC, Schwerin M. Dietary protein-related changes in hepatic transcription correspond to modifications in hepatic protein expression in growing pigs. J Nutr. 2004;134(1):43–47. 10.1093/jn/134.1.43 14704291

[44] Golovan SP, Hakimov HA, Verschoor CP, Walters S, Gadish M, Elsik C, et al Analysis of Sus scrofa liver proteome and identification of proteins differentially expressed between genders, and conventional and genetically enhanced lines. Comparative Biochemistry and Physiology Part D: Genomics and Proteomics. 2008;3(3):234–242. 10.1016/j.cbd.2008.05.001 20483222

[45] Yi S-S, Oh S-J, Kim I-Y, Yeom H-J, Yeom S-C, Hwang S-Y, et al Proteomic analysis of liver in miniature pigs according to developmental stages using two-dimensional electrophoresis and matrix-assisted laser desorption/ionization-time of flight mass spectrometry. Laboratory Animal Research. 2013;29(3):162–167. 10.5625/lar.2013.29.3.162 24106511 PMC3791350

[46] Tsujita T, Kang D, Moon MH, Ohno N, Inoue T, Matsumoto M, et al Large-Scale Identification by Shotgun Proteomics of Proteins Expressed in Porcine Liver and Salivary Gland. Zoological Science. 2008;25(2):129–138. 10.2108/zsj.25.129 18533743

[47] Liu J, Liu Z, Chen L, Zhang H. iTRAQ-based proteomic analysis reveals alterations in the liver induced by restricted meal frequency in a pig model. Nutrition. 2016;32(7–8):871–876. 10.1016/j.nut.2016.01.020 27106395

[48] Long B, Yin C, Fan Q, Yan G, Wang Z, Li X, et al Global Liver Proteome Analysis Using iTRAQ Reveals AMPK-mTOR-Autophagy Signaling Is Altered by Intrauterine Growth Restriction in Newborn Piglets. J Proteome Res. 2016;15(4):1262–1273. 10.1021/acs.jproteome.6b00001 26967195

[49] Blutke A, Renner S, Flenkenthaler F, Backman M, Haesner S, Kemter E, et al The Munich MIDY Pig Biobank–A unique resource for studying organ crosstalk in diabetes. Molecular Metabolism. 2017;6(8):931–940. 10.1016/j.molmet.2017.06.004 28752056 PMC5518720

[50] Li Y, Mou Y, Thunders M, Wu Y, Ai X, Zhou X, et al Effects of enrofloxacin on antioxidant system, microsomal enzymatic activity, and proteomics in porcine liver. J Vet Pharmacol Ther. 2018 Epub 2018/04/15. 10.1111/jvp.12493 29654626

[51] Liu Z, Yuan Y, Yin J, Li T, Zhang H, He J. Long-term effect of lysine restriction on liver global proteins, meat quality, and blood biochemical parameters in pigs. Protein Pept Lett. 2018 Epub 2018/04/07. 10.2174/0929866525666180406142451 29623821

[52] Wang J, Sun Z, Jiang J, Wu D, Liu X, Xie Z, et al Proteomic Signature of Acute Liver Failure: From Discovery and Verification in a Pig Model to Confirmation in Humans. Mol Cell Proteomics. 2017;16(7):1188–1199. 10.1074/mcp.M117.067397 28336726 PMC5500754

[53] Kampf C, Mardinoglu A, Fagerberg L, Hallstrom BM, Edlund K, Lundberg E, et al The human liver-specific proteome defined by transcriptomics and antibody-based profiling. Faseb j. 2014;28(7):2901–2914. 10.1096/fj.14-250555 24648543

[54] Miller B, Selevsek N, Grossmann J, Kilminster T, Scanlon T, Daniels M, et al Ovine liver proteome: Assessing mechanisms of seasonal weight loss tolerance between Merino and Damara sheep. Journal of Proteomics. 2018 10.1016/j.jprot.2018.02.018. 29466715

[55] Görg A, Weiss W, Dunn MJ. Current two dimensional electrophoresis technology for proteomics. Proteomics. 2004;4(12):3665 10.1002/pmic.200401031 15543535

[56] Pottiez G, Wiederin J, Fox HS, Ciborowski P. Comparison of 4-plex to 8-plex iTRAQ Quantitative Measurements of Proteins in Human Plasma Samples. Journal of Proteome Research. 2012;11(7):3774–3781. 10.1021/pr300414z 22594965 PMC3390908

[57] Bantscheff M, Schirle M, Sweetman G, Rick J, Kuster B. Quantitative mass spectrometry in proteomics: a critical review. Analytical and Bioanalytical Chemistry. 2007;389(4):1017–1031. 10.1007/s00216-007-1486-6 17668192

[58] Holmes RS, Wright MW, Laulederkind SJ, Cox LA, Hosokawa M, Imai T, et al Recommended nomenclature for five mammalian carboxylesterase gene families: human, mouse, and rat genes and proteins. Mamm Genome. 2010;21(9–10):427–441. 10.1007/s00335-010-9284-4 20931200 PMC3127206

[59] Wei E, Ben Ali Y, Lyon J, Wang H, Nelson R, Dolinsky VW, et al Loss of TGH/Ces3 in mice decreases blood lipids, improves glucose tolerance, and increases energy expenditure. Cell Metab. 2010;11(3):183–193. 10.1016/j.cmet.2010.02.005 20197051

[60] Lian J, Wei E, Wang SP, Quiroga AD, Li L, Di Pardo A, et al Liver specific inactivation of carboxylesterase 3/triacylglycerol hydrolase decreases blood lipids without causing severe steatosis in mice. Hepatology. 2012;56(6):2154–2162. 10.1002/hep.25881 22707181

[61] Parini P, Davis M, Lada AT, Erickson SK, Wright TL, Gustafsson U, et al ACAT2 is localized to hepatocytes and is the major cholesterol-esterifying enzyme in human liver. Circulation. 2004;110(14):2017–2023. 10.1161/01.CIR.0000143163.76212.0B 15451793

[62] Hai Q, Ritchey B, Robinet P, Alzayed AM, Brubaker G, Zhang J, et al Quantitative Trait Locus Mapping of Macrophage Cholesterol Metabolism and CRISPR/Cas9 Editing Implicate an ACAT1 Truncation as a Causal Modifier Variant. Arterioscler Thromb Vasc Biol. 2018;38(1):83–91. 10.1161/ATVBAHA.117.310173 29097366 PMC5746475

[63] Wang Z, Shang P, Li Q, Wang L, Chamba Y, Zhang B, et al iTRAQ-based proteomic analysis reveals key proteins affecting muscle growth and lipid deposition in pigs. Scientific Reports. 2017;7:46717 10.1038/srep46717 28436483 PMC5402282

[64] Fontanesi L, Schiavo G, Galimberti G, Bovo S, Russo V, Gallo M, et al A genome-wide association study for a proxy of intermuscular fat level in the Italian Large White breed identifies genomic regions affecting an important quality parameter for dry-cured hams. Anim Genet. 2017;48(4):459–465. 10.1111/age.12542 28696026

[65] Zhang W, Bin Y, Zhang J, Cui L, Ma J, Chen C, et al Genome-wide association studies for fatty acid metabolic traits in five divergent pig populations. Sci Rep. 2016;6:24718 10.1038/srep24718 27097669 PMC4838829

[66] Marzluff WF, Gongidi P, Woods KR, Jin J, Maltais LJ. The Human and Mouse Replication-Dependent Histone Genes. Genomics. 2002;80(5):487–498. 12408966

[67] Drab T, Kracmerova J, Hanzlikova E, Cerna T, Litvakova R, Pohlova A, et al The antimicrobial action of histones in the reproductive tract of cow. Biochem Biophys Res Commun. 2014;443(3):987–990. 10.1016/j.bbrc.2013.12.077 24361880

[68] Li GH, Mine Y, Hincke Maxwell T, Nys Y. Isolation and characterization of antimicrobial proteins and peptide from chicken liver. Journal of Peptide Science. 2007;13(6):368–378. 10.1002/psc.851 17431854

[69] Noga EJ, Borron PJ, Hinshaw J, Gordon WC, Gordon LJ, Seo J-K. Identification of histones as endogenous antibiotics in fish and quantification in rainbow trout (Oncorhynchus mykiss) skin and gill. Fish Physiology and Biochemistry. 2011;37(1):135–152. 10.1007/s10695-010-9422-7 20711849

[70] Henryon M, Berg P, Jensen J, Andersen S. Genetic variation for resistance to clinical and subclinical diseases exists in growing pigs. Animal Science. 2001;73(3):375–387.

