# Supplementary material for: A comparative analysis of label-free liquid chromatography-mass spectrometry liver proteomic profiles highlights metabolic differences between pig breeds
Source: PLoS One. 2018 Sep 12;13(9):e0199649. doi: 10.1371/journal.pone.0199649 (PMC6135354; doi:10.1371/journal.pone.0199649)
Supplement: S3 Table — (DOCX) [file pone.0199649.s003.docx]

**S3 Table. Links between differentially expressed proteins identified in the comparison between breeds and QTLs mapped in the corresponding gene regions (± 50 kbp).**

| **Differentially abundant protein^1^** | | | | **Quantitative Trait Locus^2^** | | | | | |  |  |
| --- | --- | --- | --- | --- | --- | --- | --- | --- | --- | --- | --- |
| **Protein** | **Chr** | **Start** | **End** | **QTL** | **Chr** | **Start** | **End** | **Distance (bp)** | **Protein Location** | **Direction^3^** | **Breed** |
| ECHDC1 | 1 | 35852494 | 35911541 | Saturated fatty acid content QTL | 1 | 35898153 | 36238562 | - | Within | IDU | Duroc pigs |
| ECHDC1 | 1 | 35852494 | 35911541 | body weight (60 days) QTL | 1 | 35898153 | 36238562 | - | Within | IDU | F2 from crossing Dabai and Meishan pigs |
| PRDX5 | 2 | 7798670 | 7802043 | backfat at mid-back QTL | 2 | 7804743 | 150596593 | 2700 | Upstream | ILW | Animals were a cross between Large White and Chinese Meishan |
| PRDX5 | 2 | 7798670 | 7802043 | Body weight QTL | 2 | 7816376 | 8537748 | 14333 | Upstream | ILW | Animals were purebred and crossbred pigs representing several breeds: Duroc, Cerdo Iberico, Large Black, Hampshire, Pietrain, and Schwabisch-Hallisch, and European Wild Boar) |
| SPR | 3 | 69887386 | 69895384 | CD8-negative leukocyte percentage QTL | 3 | 69920446 | 69920486 | 25062 | Upstream | ILW | Animals were from a Duroc x Erhualian F2 resource population |
| SPR | 3 | 69887386 | 69895384 | CD3-negative, CD8-negative leukocyte percentage QTL | 3 | 69920446 | 69920486 | 25062 | Upstream | ILW | Animals were from a Duroc x Erhualian F2 resource population |
| SPR | 3 | 69887386 | 69895384 | CD8-positive leukocyte percentage QTL | 3 | 69920446 | 69920486 | 25062 | Upstream | ILW | Animals were from a Duroc x Erhualian F2 resource population |
| SPR | 3 | 69887386 | 69895384 | CD8-negative leukocyte percentage QTL | 3 | 69935625 | 69935665 | 40241 | Upstream | ILW | Animals were from a Duroc x Erhualian F2 resource population |
| SPR | 3 | 69887386 | 69895384 | CD3-negative, CD8-negative leukocyte percentage QTL | 3 | 69935625 | 69935665 | 40241 | Upstream | ILW | Animals were from a Duroc x Erhualian F2 resource population |
| SPR | 3 | 69887386 | 69895384 | CD8-positive leukocyte percentage QTL | 3 | 69935625 | 69935665 | 40241 | Upstream | ILW | Animals were from a Duroc x Erhualian F2 resource population |
| KHK | 3 | 112021165 | 112031620 | Cholesterol level QTL | 3 | 111990482 | 112135038 | 30683 | Downstream | ILW | commercial Duroc line |
| CES3 | 6 | 27627759 | 27637508 | Residual feed intake QTL | 6 | 27650977 | 28138132 | 13469 | Upstream | ILW | Large White pigs |
| HSD17B14 | 6 | 54125407 | 54143532 | Interleukin 10 level QTL | 6 | 54156624 | 54156664 | 13092 | Upstream | IDU | Animals were Landrace, Yorkshire, and Songliao Black pigs |
| HSD17B14 | 6 | 54125407 | 54143532 | Interleukin 10 level QTL | 6 | 54103234 | 54103274 | 22133 | Downstream | IDU | Animals were Landrace, Yorkshire, and Songliao Black pigs |
| GSTA1 | 7 | 46538613 | 46588218 | Backfat at rump QTL | 7 | 45927708 | 46490681 | 47932 | Downstream | ILW | Animals were from a series of crosses consisting of Large White, Meishan, and Landrace |
| GSTA1 | 7 | 46538613 | 46588218 | Feed conversion ratio QTL | 7 | 45927708 | 46490681 | 47932 | Downstream | ILW | Italian Large White pigs |
| GSTA1 | 7 | 46538613 | 46588218 | Backfat at last rib QTL | 7 | 45927708 | 46490681 | 47932 | Downstream | ILW | Animals were from a series of crosses consisting of Large White, Meishan, and Landrace |
| GSTA1 | 7 | 46538613 | 46588218 | Backfat between 3rd and 4th last ribs QTL | 7 | 45927708 | 46490681 | 47932 | Downstream | ILW | Animals were from a series of crosses consisting of Large White, Meishan, and Landrace |
| GSTA1 | 7 | 46538613 | 46588218 | Backfat weight QTL | 7 | 45927708 | 46490681 | 47932 | Downstream | ILW | Animals were from a series of crosses consisting of Large White, Meishan, and Landrace |
| GSTA1 | 7 | 46538613 | 46588218 | Abdominal fat weight QTL | 7 | 41308920 | 46490681 | 47932 | Downstream | ILW | Animals were from a cross between Chinese Erhualian and White Duroc pigs |
| GSTA1 | 7 | 46538613 | 46588218 | backfat between the last 3rd and 4th lumbar QTL | 7 | 45927708 | 46490681 | 47932 | Downstream | ILW | Animals were from a series of crosses consisting of Large White, Meishan, and Landrace |
| GSTA1 | 7 | 46538613 | 46588218 | Shoulder subcutaneous fat thickness QTL | 7 | 45927708 | 46490681 | 47932 | Downstream | ILW | Animals were from a series of crosses consisting of Large White, Meishan, and Landrace |
| GSTA1 | 7 | 46538613 | 46588218 | Average daily gain QTL | 7 | 45927708 | 46490681 | 47932 | Downstream | ILW | Animals were from a series of crosses consisting of Large White, Meishan, and Landrace |
| GSTA1 | 7 | 46538613 | 46588218 | Unsaturated fatty acid content QTL | 7 | 45927708 | 46490681 | 47932 | Downstream | ILW | Animals were a White Duroc x Erhualian cross |
| GSTA1 | 7 | 46538613 | 46588218 | Linolenic acid content QTL | 7 | 45927708 | 46490681 | 47932 | Downstream | ILW | Animals were a White Duroc x Erhualian cross |
| GSTA1 | 7 | 46538613 | 46588218 | Monounsaturated fatty acid content QTL | 7 | 45927708 | 46490681 | 47932 | Downstream | ILW | Animals were a White Duroc x Erhualian cross |
| GSTA1 | 7 | 46538613 | 46588218 | Oleic acid content QTL | 7 | 45927708 | 46490681 | 47932 | Downstream | ILW | Animals were a White Duroc x Erhualian cross |
| FAH | 7 | 49047983 | 49087783 | Backfat at rump QTL | 7 | 49051177 | 49316513 | - | Within | IDU | crosses consisting of Large White, Meishan, and Landrace |
| FAH | 7 | 49047983 | 49087783 | Carcass length QTL | 7 | 49051177 | 49316513 | - | Within | IDU | crosses consisting of Large White, Meishan, and Landrace |
| FAH | 7 | 49047983 | 49087783 | Average glycolytic potential QTL | 7 | 48798335 | 49051177 | - | Within | IDU | White Duroc x Erhualian |
| FAH | 7 | 49047983 | 49087783 | Average backfat thickness QTL | 7 | 49051177 | 49316513 | - | Within | IDU | Crosses consisting of Large White, Meishan, and Landrace |
| FAH | 7 | 49047983 | 49087783 | Cis-11-Eicosenoic acid to oleic acid ratio QTL | 7 | 49071361 | 49071401 | - | Within | IDU | Animals were from five pig populations: White Duroc x Erhualian F2, Duroc x (Landrace x Yorkshire) hybrid, Sutai, Erhualian, and Laiwu. |
| FAH | 7 | 49047983 | 49087783 | Intramuscular fat content QTL | 7 | 49132319 | 49132359 | 44536 | Upstream | IDU | inbred Berkshire population |
| UROC1 | 7 | 53590895 | 53637709 | Eicosadienoic acid to linoleic acid ratio QTL | 7 | 53559750 | 53559790 | 31105 | Downstream | IDU | Animals were from five pig populations: White Duroc x Erhualian F2, Duroc x (Landrace x Yorkshire) hybrid, Sutai, Erhualian, and Laiwu. |
| UROC1 | 7 | 53590895 | 53637709 | Cis-11-Eicosenoic acid to oleic acid ratio QTL | 7 | 53559750 | 53559790 | 31105 | Downstream | IDU | Animals were from five pig populations: White Duroc x Erhualian F2, Duroc x (Landrace x Yorkshire) hybrid, Sutai, Erhualian, and Laiwu |
| ACAT1 | 9 | 36525261 | 36545633 | Intramuscular fat content QTL | 9 | 36476098 | 36476138 | 49123 | Downstream | IDU | Inbred Berkshire population |
| FASN | 12 | 922418 | 937560 | Myristic acid content QTL | 12 | 940527 | 940567 | 2967 | Upstream | ILW | Duroc pigs. |
| FASN | 12 | 922418 | 937560 | Palmitic acid to myristic acid ratio QTL | 12 | 940527 | 940567 | 2967 | Upstream | ILW | Animals were from five pig populations: White Duroc x Erhualian F2, Duroc x (Landrace x Yorkshire) hybrid, Sutai, Erhualian, and Laiwu |
| FASN | 12 | 922418 | 937560 | Daily feed intake QTL | 12 | 79502 | 980500 | - | Within | ILW | Boars were from the terminal Maxgro line, which is predominately Pietrain based |
| CPS1 | 15 | 113209631 | 113334672 | Estimated carcass lean content QTL | 15 | 112846305 | 113312193 | - | Within | IDU | Canadian three-way cross |

^1^Genome coordinates are based on the *S. scrofa* v.11.1 reference genome; ^2^QTL information are retrieved from the Pig QTLdb resource; ^3^“ID” indicates protein abundance higher in Italian Duroc pigs than Italian Large White pigs while ‘ILW’ indicates protein abundance higher in Italian Large White pigs than Italian Duroc pigs.
